# Smoking as a Risk Factor for Dry Socket: A Systematic Review

**DOI:** 10.3390/dj10070121

**Published:** 2022-07-01

**Authors:** Weronika Kuśnierek, Kaja Brzezińska, Kacper Nijakowski, Anna Surdacka

**Affiliations:** 1 Student’s Scientific Group in Department of Conservative Dentistry and Endodontics, Poznan University of Medical Sciences, 60-812 Poznan, Poland; wera1808@wp.pl (W.K.); kajaa.brzezinska@gmail.com (K.B.); 2 Department of Conservative Dentistry and Endodontics, Poznan University of Medical Sciences, 60-812 Poznan, Poland

**Keywords:** dry socket, alveolitis, smoking, cigarettes, tooth extraction, dental surgery

## Abstract

Dry socket is one of the postoperative complications of tooth extraction. It is the partial or total loss of the post-extraction blood clot, resulting in severe pain that usually starts one to five days postoperatively, with clinical evidence of exposed alveolar bone, necrotic debris, halitosis, and tenderness on examination. The purpose of our systematic review was to answer the question “Is there a relationship between smoking and dry socket?”. After meeting the inclusion and exclusion criteria, eleven studies were included in this systematic review (according to the PRISMA statement guidelines). Based on a meta-analysis, tobacco smokers had a more than three-fold increase in the odds of dry socket after tooth extraction. Overall, the combined incidence of dry socket in smokers was found to be about 13.2% and in non-smokers about 3.8%. Despite the heterogeneity of the included studies (different types of teeth extracted, different age groups), cigarette smoking was related to an increased risk of dry socket after tooth extraction.

## 1. Introduction

Tooth extraction is one of the most frequent procedures in surgical dentistry, particularly widespread during the first wave of the COVID-19 pandemic [1,2]. The indications include oral mucositis, periapical inflammation, bone loss, periodontal ligament damage, orthodontic indications, resorption of adjacent teeth [3,4]. Extraction techniques are divided into nonsurgical extractions and surgical extractions. Nonsurgical extractions require simple elevation or separation of the root without reflection of the mucoperiosteal flap. Surgical extractions need the reflection of the mucoperiosteal flap with or without bone removal [5,6]. Tooth extraction is associated with complications both during and after the procedure [7]. The most common complications are prolonged bleeding, purulent alveolitis, postoperative pain, and dry socket [8].

As mentioned above, one of the more common complications is dry socket [9]. Dry socket is the partial or total loss of the post-extraction blood clot. It usually starts with severe pain one to five days postoperatively, what is accompanied by clinical symptoms such as exposed alveolar bone, necrotic debris, and halitosis. Other terms for this condition include alveolar osteitis, fibrinolytic alveolitis, localised osteomyelitis, postoperative alveolitis, and alveolitis sicca [10]. The pathogenesis of dry socket remains still unclear [11]. The most popular theory is the disintegration of the blood clot in the alveolus caused by elevated fibrinolytic activity. The initiation of the fibrinolysis is reported to be associated with several factors such as the following: age, gender, smoking habits, oral contraceptives use, menstrual cycle, surgery duration, surgical trauma, condition of the extracted teeth, type of the extracted teeth, presence of a previous periapical or pericoronal infection, inadequate curettage or irrigation of the socket after extraction, excessive use of local anaesthetic with vasoconstrictor [12].

The adverse effects of cigarette smoking on oral health are well established, extending from cosmetic effects, such as tooth staining, to potentially life-threatening conditions such as oral cancer [13]. Particularly, pathological effects of smoking on periodontal tissues that render smokers more susceptible to periodontal disease have been reviewed in the literature [14,15]. Previous studies present that smokers demonstrate a higher prevalence, severity, and progression of periodontitis compared to former smokers or non-smokers. Moreover, tobacco smoking negatively influences nonsurgical and surgical periodontal therapy, including regenerative and plastic surgeries. Although heavy cigarette smokers suffer from more severe forms of periodontitis and unfavourable treatment prognosis, quitting smoking decreases the progression of periodontal destruction and leads to better clinical outcomes [16]. The systematic review by Ralho et al. [17] suggests that the use of electronic cigarettes is less harmful to oral health. However, the authors observed a greater susceptibility to developing different lesions of the oral mucosa than ex-smokers or non-smokers.

Our systematic review was designed in order to answer the question “Is there a relationship between smoking and dry socket?”, formulated according to PICO (population, intervention, comparison, and outcome).

## 2. Materials and Methods

### 2.1. Search Strategy and Data Extraction

A systematic review was conducted up to 10th March 2022, according to the preferred reporting items for systematic reviews and meta-analyses (PRISMA) statement guidelines [18], using the databases PubMed, Scopus, and Web of Science. The search formulas included:

- For PubMed: ((dry socket) OR (dry alveol*) OR (alveolar osteitis) OR (alveolitis osteitis) OR (fibrynolitic alveolitis)) AND ((smoker*) OR (alcoholic*) OR (smoking) OR (alcohol consumption) OR (cigarette) OR (nicotine) OR (tobacco) OR (alcohol) OR (alcohol addiction) OR (cigarette addiction))
- For Scopus: TITLE-ABS-KEY ((“dry socket”) OR (“dry alveol*”) OR (“alveolar osteitis”) OR (“alveolitis osteitis”) OR (“fibrynolitic alveolitis”)) AND ((smoker*) OR (alcoholic*) OR (smoking) OR (“alcohol consumption”) OR (cigarette) OR (nicotine) OR (tobacco) OR (alcohol) OR (“alcohol addiction”) OR (“cigarette addiction”))
- For Web of Science: TS = (dry socket OR dry alveol* OR alveolar osteitis OR alveolitis osteitis OR fibrynolitic alveolitis) AND TS = (smoker* OR alcoholic* OR smoking OR alcohol consumption OR cigarette OR nicotine OR tobacco OR alcohol OR alcohol addiction OR cigarette addiction).

The results were filtered by publication date (after 2000).

Records were screened by the title, abstract, and full text by two independent investigators. Studies included in this review matched all the predefined criteria according to PICOS (population, intervention, comparison, outcome, and study design), as shown in Table 1. A detailed search flowchart is presented in the Results section. The study protocol was registered in the international prospective register of systematic reviews PROSPERO (CRD42022336307).

The results of the meta-analysis are presented in forest plots using MedCalc Statistical Software version 19.5.3 (MedCalc Software Ltd., Ostend, Belgium) and Statistica 13.3 software (StatSoft, Cracow, Poland).

### 2.2. Quality Assessment and Critical Appraisal for the Systematic Review of Included Studies

The risk of bias in each individual study was assessed according to the study quality assessment tool issued by the National Heart, Lung, and Blood Institute within the National Institute of Health [19]. These questionnaires were answered by two independent investigators, and any disagreements were resolved by discussion between them.

**Figure 1:**
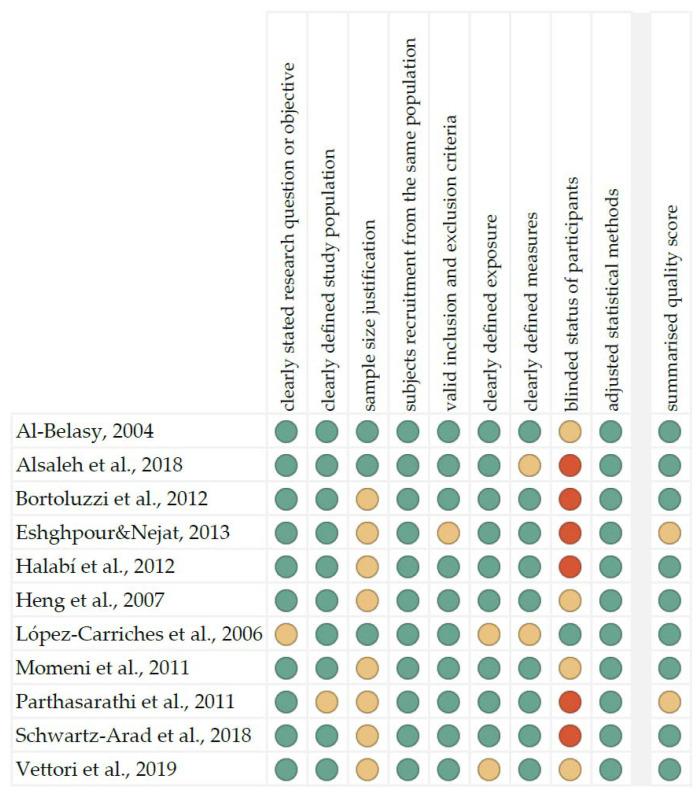
Quality assessment, including the main potential risk of bias (risk level: green—low, yellow—unspecified, red—high; quality score: green—good, yellow—intermediate, red—poor) [21,22,23,24,25,26,27,28,29,30,31].

The summarised quality assessment for every single study is reported in Figure 1. Critical appraisal was summarised by adding up the points for each criterion of potential risk (points: 1—low, 0.5—unspecified, 0—high). Nine studies (81.8%) were classified as having “good” quality (≥80% total score) and two (18.2%) as “intermediate” (≥60% total score).

The level of evidence was assessed using the classification of the Oxford Centre for Evidence-Based Medicine levels for diagnosis [20]. All of the included studies have the third or fourth level of evidence (in this 5-graded scale).

## 3. Results

**Figure 2:**
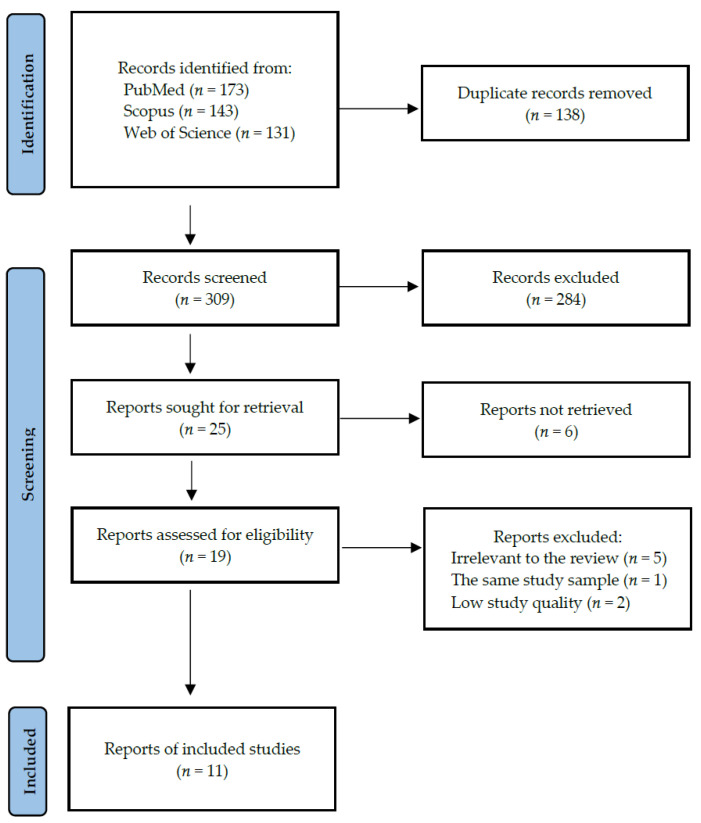
PRISMA flow diagram presenting search strategy.

Following the search criteria, our systematic review included eleven studies, demonstrating data collected in ten different countries from a total of 10195 participants (including 3007 smokers and 7188 non-smokers). Figure 2 shows the detailed selection strategy of the articles. The inclusion and exclusion criteria are presented in Table 1 (in the Materials and Methods section).

From each eligible study included in the present systematic review, we collected data about its general characteristics, such as year of publication and setting, involved participants (gender and age), smoking status, inclusion and exclusion criteria, medical history, oral hygiene (Table 2). Table 3 presents the detailed characteristics considering prevalence of dry socket, kind of extracted tooth, extraction technique, symptoms recognised as the onset of dry socket, and provided prophylaxis or treatment.

**Figure 3:**
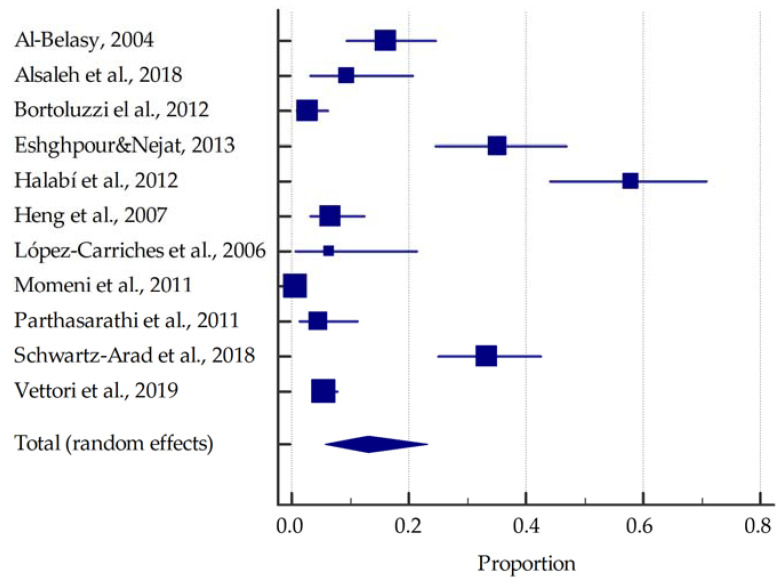
Forest plot presenting the summarised prevalence of dry socket among smokers [21,22,23,24,25,26,27,28,29,30,31].

**Figure 4:**
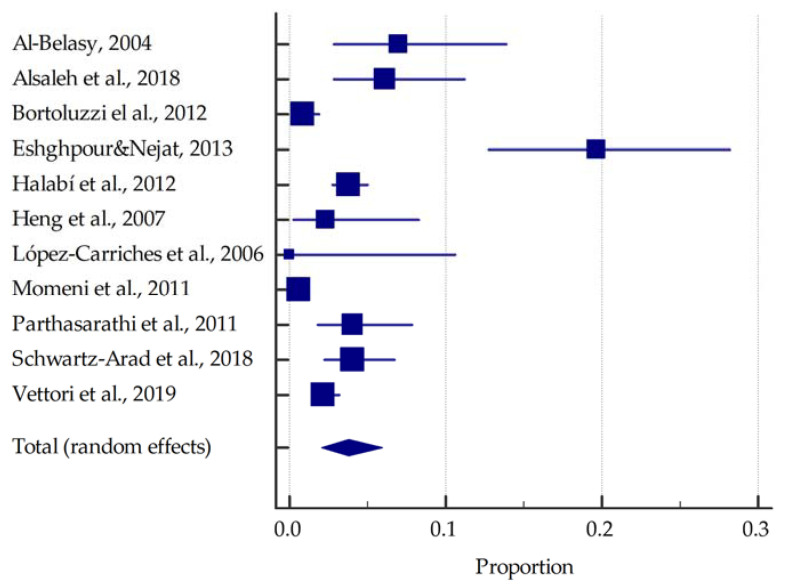
Forest plot presenting the summarised prevalence of dry socket among non-smokers [21,22,23,24,25,26,27,28,29,30,31].

On the basis of the included studies reporting the prevalence of dry socket in smokers, it was determined that the summarised prevalence is approximately 13.2% (95%CI: 5.8–23.1%) (Figure 3). In contrast, in non-smokers the summarised prevalence of dry socket was estimated to be around 3.8% (95%CI: 2.1–6.0%) (Figure 4).

**Figure 5:**
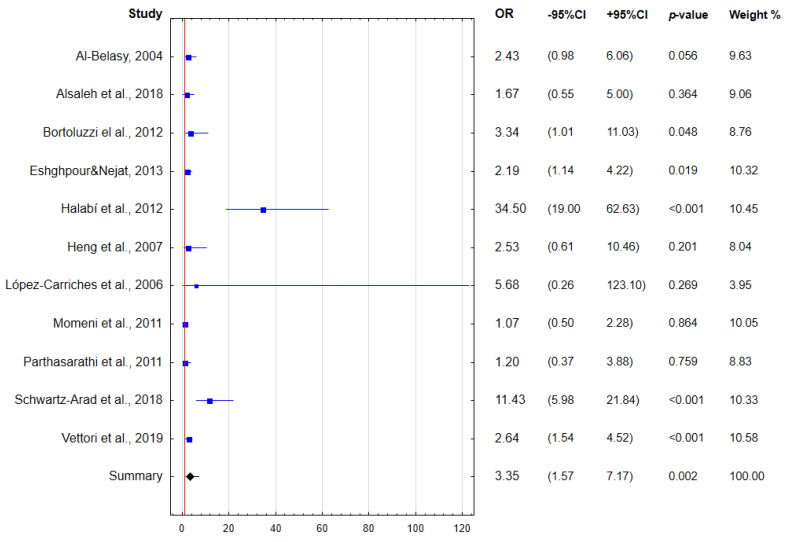
Forest plot presenting the odds for dry socket considering regular smoking (OR—odds ratio; CI—confidence interval) [21,22,23,24,25,26,27,28,29,30,31].

Altogether, regular smoking is associated with a more than three-fold increase in the odds of dry socket after tooth extraction (Figure 5).

## 4. Discussion

Our systematic review found an association between the dry socket occurrence and cigarette smoking. Because of the type of extraction (surgical/nonsurgical), type of tooth extracted, and amount of cigarette smoking, it is difficult to relate the summarised results to individual studies. Therefore, the literature discussed sequentially begins with simple extractions of all kinds and ends with the literature about only retained third molars. An additional criterion for the studies discussed was the number of cigarettes smoked.

Halabi et al. [25] presented a logistic regression analysis of risk factors for the alveolitis development. Prior extraction site infection, surgical trauma, and smoking habits were associated with an increased risk of dry socket. They included 1302 participants who underwent tooth extractions in their study. Extractions were performed on all types of teeth. Tobacco use was assessed as smoking (above 5 cigarettes 24 h after extraction) or non-smoking (below 5 cigarettes 24 h after extraction). The incidence of alveolar osteitis was 6.8% in patients overall. However, in smokers, the dry socket incidence was 58%.

In the most recent study, Vettori et al. [31] discussed the factors affecting the occurrence of intraoperative and postoperative complications after tooth extraction: a retrospective study on a group of 1701 patients. They extracted every type of tooth. This study aimed to evaluate the type and frequency of complications after exodontic procedures, their correlation with antibiotic administration and with patient-related systemic factors. Smoking habits and presented coagulopathy were associated with higher risk of postoperative alveolitis. Based on the results presented here, antibiotic intake did not appear to reduce the incidence of postoperative infectious complications.

Bortoluzzi et al. [23] attempted to answer whether smoking increases the incidence of postoperative complications after simple extractions. They conducted a single-centre, prospective study of postoperative complications in the alveolar process. Excluded from the study were extractions of third molars that had not fully erupted or were classified as difficult to remove by students, as well as extractions of deciduous teeth. Logistic regression showed that tooth dissection, smoking, and the number of cigarettes smoked (>20 cigarettes per day) were associated with the occurrence of dry socket.

In contrast, Parthasaranthi et al. [29] obtained different results from the other authors. They discussed several factors that could be associated with the occurrence of dry socket. In their study, they examined tooth extractions of all types. Logistic regression analysis showed that posterior teeth, teeth extracted due to periapical disease, intraoperative crown-root fractures, teeth extracted by specialists or dentists, and psychotropic medication history were significant independent factors for the alveolar osteomyelitis development. However, the most important finding of the study was that smoking habits and extraction techniques (nonoperative or operative) were not found to significantly influence the alveolitis development.

Momeni et al. [28] evaluated distribution and risk factors in patients with dry socket referring to their dental clinics after extractions of all types of teeth. The results showed that alveolitis was more common in women than in men. The ratio of mandible to maxilla was 2.5 to 1, and mandibular third molars were more often involved than other teeth. Smoking habits, poor oral hygiene, and surgical trauma increased the dry socket incidence

Similarly, Alshaleh et al. [22] discussed how patients’ behaviours after tooth extraction affect the development of dry socket. For their study, they did not consider retained third molars. Among other things, 26.4% of the patients were smokers. These participants were instructed not to smoke for the next three days, some of them complied, and some did not. In total, 68% of the smokers smoked on the extraction day, and the rest did not smoke (for the next 72 h). There was no significant association between smokers and patients who developed dry alveolus.

Heng et al. [26] examined the relationship between cigarette smoking and dry socket incidence in the total number of extractions, only for third molar extractions. They found a significant difference in the incidence of overall complications between smokers and non-smokers. There was a significant difference in the incidence of alveolar osteitis between mandibular third molar extractions and other extractions regardless of smoking status. Moreover, surgical trauma contributed significantly to the increase in overall complications and alveolar osteitis, as well as smoking seemed to be a causative factor to the increase in complications among multiple extractions. In this study, smoking habits, mandibular third molars, and surgical trauma were significantly related to higher rates of postoperative complications, including alveolitis.

The first study that focused on the lower third molars was conducted by Lopez-Carriches et al. [27]. Although two cases of alveolitis were documented among smokers, there was no statistically significant difference between these groups in pain complaints, but trismus was higher in smokers. Smoking habits had no effect on wound condition.

Another study focusing on third molars was performed in an Iranian population by Eshghpour and Nejat [24]. They found that some factors such as gender, systemic diseases, age, and use of antibiotics before surgery showed no significant association with the alveolitis incidence. However, the incidence of dry socket was significantly associated with smoking. In addition to smoking, other factors associated with the incidence of dry alveolus were: the difficulty of the surgery, length of the surgery, oral contraceptives use, menstrual cycle, and number of carpules used to achieve anaesthesia.

Al-Belasy [21] also focused on the same type of teeth as the previous article mentioned above. His study found that smokers had a two- to three-fold higher risk of dry socket than non-smokers. Patients who smoked on the day of surgery had a significantly higher incidence of dry socket than ones who smoked on the second day after surgery. Increased frequency of smoking and smoking on the day of surgery significantly elevated the prevalence of dry socket.

Lastly, Schwartz-Arad et al. [30] analysed the incidence of complications after third molar extraction according to risk factors. The most common complication in this study was dry socket. Partially retained teeth had the highest incidence of complications. Cigarette smoking was associated with a higher incidence of dry sockets, and complications were more common on the left side. The authors concluded that complications after mandibular third molar surgical removal increase with smoking habits, age, degree of enucleation, and extraction site.

The practical value of our systematic review should be emphasised, confirming the potential relationship between the negative habit of smoking by patients and the occurrence of complications after surgical procedures, such as dry socket. From a clinical point of view, the obtained results oblige dentists to intensify pro-health education of patients in order to avoid the harmful effects of the cigarettes. The conducted systematic review had limitations related to the heterogeneity of the included studies. The main reasons of potential bias include different age groups of patients, gender, and racial diversity, as well as different tooth extraction techniques and types of extracted teeth. Moreover, the selected research design models and the various sizes of the studied samples were not without significance. Therefore, further prospective studies on larger groups of patients are necessary, taking into account in detail the frequency of smoking, the use of other stimulants or the presence of comorbidities.

## 5. Conclusions

Cigarette smoking is related to an increased risk of dry socket. Our review found that approximately 13.2% of cigarette smokers developed a dry socket after tooth extraction. However, it is difficult to establish clear associations due to the heterogeneity of the included studies (different types of extracted teeth, different age groups).

## Figures and Tables

**Table 1:** Inclusion and exclusion criteria according to the PICOS.

Parameter	Inclusion Criteria	Exclusion Criteria
Population	patients with dry socket—both genders, regardless of age	patients with other complications after tooth extraction
Intervention	smoking	
Comparison	non-smoking	
Outcomes	prevalence of dry socket	prevalence of dry socket with other predisposing factors, such as alcohol, contraceptives, or water pipe
Study design	case-control, cohort and cross-sectional studies	literature reviews, case reports, expert opinion, conference reports
	published after 2000	not published in English

**Table 2:** General characteristics of included studies.

Author, Year, Setting	Participants (F/M)	Age (Years)	Smoking Status (% of Smokers)	Inclusion Criteria	Exclusion Criteria	Comorbidities	Oral Hygiene Status
Al-Belasy, 2004, Egypt [21]	200 (0/100)	mean 27 (range: 20–38)	50.0	patients who were treated at the Oral Surgery Department, Faculty of Dentistry, Mansoura University between January 2000 and February 2002, healthy patients required to have unilateral high mesioangular impactions of a mandibular third molar with an exposed occlusal surface	women, former smokers, men who smoked both cigarettes and shisha, patients with recent antibiotic use, and patients with medical need for prophylactic antibiotics	100% no systemic disease	NR
Alsaleh et al., 2018 Kingdom of Saudi Arabia [22]	201 (79/122)	NR	26.4	patients classified as ASA I (healthy patients) and ASA II (patients with mild, controlled systemic disease without functional limitation), patients with a history of nonsurgical extraction of a permanent tooth	patients who required treatment under general anaesthesia, children under 6 years of age who have not yet grown permanent teeth, and all patients with exodontia of primary teeth and retained teeth	90.1% no systemic disease	NR
Bortoluzzi et al., 2012, Brazil [23]	793 (337/456)	41.6 ± 16.0 (range: 9–85)	23.3	simple and erupted teeth exodontia, procedures conducted by undergraduate students under similar conditions between March 2007 and December 2011	extractions of third molars that had not fully erupted and/or were classified as difficult for undergraduate students to remove and extractions of deciduous teeth	NR	NR
Eshghpour & Nejat, 2013, Iran [24]	189 (91/98)	18–48	40.7	extraction of impacted third mandibular molar teeth performed between April 2009 and August 2010 in Dental Clinic of Oral and Maxillofacial Surgery	NR	86.0% no systemic disease	prior to surgery, all the patients underwent a thorough scaling and oral prophylaxis
Halabí et al., 2012, Chile [25]	1302 (90/1212)	39.7 ± 16	4.4	patients who underwent dental extraction from March to June 2011 in dental clinic in Valdiva, Chile	extraction in the operating theatre necessary, residents of rural areas who did not present themselves for the follow-up, patients undergoing antimicrobial therapy	96.8% no systemic disease	8% poor oral hygiene
Heng et al., 2007 USA [26]	219 (219/0)	mean 37.7	61.1	inmates who had tooth extractions in the 8 months before the smoking ban (January 2004–August 2004) and 8 months after the ban (September 2004–April 2005)	inmates whose tooth extractions were performed at different times	NR	NR
López-Carriches et al., 2006, Spain [27]	64 (46/18)	mean 23.5 (range: 18–53)	48.4	patients subjected to lower third molar extraction in the Unit of Oral and Maxillofacial Surgery (Madrid Complutense University, Spain), healthy volunteers over age 18 years and requiring surgical lower third molar extraction, absence of systemic disease, absence of any habitual medication	pregnant or nursing women, allergy to local anaesthetics, antibiotics, or analgesics, patients with cardiovascular disease or any other systemic pathology	100% no systemic disease	68.8% of the patients claimed not to have brushed in the zone at the time of suture removal
Momeni et al., 2011 Iran [28]	4779 (2197/2581)	with dry socket 36.61 ± 13.59, without dry socket 42.86 ± 15.49	34.7	patients referred to dental clinics in Yazd for tooth extraction between May 2010 and June 2010	patients referred to dental clinics in Yazd for tooth extraction in another time period	63.7% no systemic disease	64% poor oral hygiene
Parthasarathi et al., 2011, Australia [29]	284 (142/142)	NR	30.8	patients having an exodontia procedure at 4 comparable public dental clinics in Victoria between June and September 2008	patients who underwent an exodontic procedure at 4 comparable public dental clinics in Victoria during a different time period	47.0% no systemic disease	85.3% poor oral hygiene
Schwartz-Arad et al., 2018, Israel [30]	463 (257/206)	mean 29 (range: 13–75)	26.0	patients having third molar extractions at Schwartz Arad Surgical Center between 2001 and 2011	patients having extractions of a tooth other than a third molar	NR	NR
Vettori et al., 2019, Italy [31]	1701 (845/876)	55.3 ± 19.9	29.7	patients who underwent single or multiple tooth extractions between June 2015 and February 2016 at the University of Trieste	patients subjected to periodontal surgery or major oral surgery, patients without specification of which antibiotic was prescribed after extraction	40.0% no systemic disease	caries was the reason of 57% extractions and periodontitis was of 31%

Legend: F, females; M, males; NR, not reported; ASA, American Society of Anaesthesiologists; USA, the United States of America.

**Table 3:** Detailed characteristics of included studies considering prevalence of dry socket.

Study	Prevalence of Dry Socket in All Patients [%]	Prevalence of Dry Socket in Smokers [%]	Tooth Extracted	Extraction Technique	Symptoms Recognised as the Onset of Dry Socket	Provided Prophylaxis or Treatment
Al-Belasy, 2004 [21]	11.5	16.0	100% impacted mandibular third molars	100% atraumatic extractions	constant radiating pain not relieved by the analgesic, accompanied by a denuded socket or necrotic clot and a fetid smell	postoperative medications given orally for analgesia were naproxen or diflunisal at a dose of 500 mg twice daily; if dry socket was diagnosed, sockets were irrigated with saline and packed with a eugenol-iodoform dressing
Alsaleh et al., 2018 [22]	7.0	9.4	all teeth except retained third molars	single tooth extractions	severe pain at the extraction site within 3 days, no blood clot at the extraction site, visible bone at the extraction site, bad breath, bad taste in mouth	patients were given post-extraction instructions verbally after the extraction
Bortoluzzi et al., 2012 [23]	1.3	2.7	all kinds of fully erupted teeth	12% traumatic extractions, 88% simple extractions	NR	NR
Eshghpour & Nejat, 2013 [24]	25.9	35.1	100% impacted mandibular third molars	100% traumatic extractions	1 to 3 days after extraction with severe pain, halitosis, foul taste, and regional lymphadenitis; no blood clot in the socket and the bone is exposed	flap sutured using 3-0 silk suture; regimen of amoxicillin (500 mg) and Gelofen (400 mg cap, TID, for maximum 3 days) was prescribed
Halabí et al., 2012 [25]	6.1	57.9	93.6% maxillary, 6.4% mandibular	4.9% traumatic extractions, 95.1% simple extractions	increasing postoperative pain intensity for 4 days within and around the socket and/or total or partial breakdown of the blood clot in the socket with or without bone exposure	reported measures for alleviating alveolar osteitis in high-risk patients include local treatment with tetracycline or preoperative and 7-day postoperative rinsing with 0.12% chlorhexidine
Heng et al., 2007 [26]	5.0	6.7	83.1% maxillary, 16.9% mandibular	27.9% traumatic extractions, 72.1% simple extractions	alveolar osteitis, pain, swelling, bleeding	patients received a verbal and written postoperative recommendation, usually ibuprofen as an analgesic; for postoperative complaints, patients had open access to the clinic
López-Carriches et al., 2006 [27]	3.1	6.5	100% lower third molar	NR	wound appearance and condition were assessed in terms of colour, marginal swelling, ulceration, the presence of plaque	no antibiotic treatment was prescribed postoperatively, and the patients received only metamizole as analgesic treatment, diclofenac was also prescribed as antiinflammatory treatment
Momeni et al., 2011 [28]	0.6	0.6	36.3% maxillary, 63.7% mandibular	NR	throbbing pain, oral malodour, and unpleasant taste; onset of symptoms 42–72 h after tooth extraction and there is no redness or purulent discharge at the affected sites	palliative intervention with prescribing anti-inflammatory drugs
Parthasarathi et al., 2011 [29]	4.2	4.6	38.8% maxillary, 61.2% mandibular	17% traumatic extractions, 83% simple extractions	the patient’s history of pain and the presence of exposed bone, intraorally	NR
Schwartz-Arad et al., 2018 [30]	11.7	33.3	100% third molar extraction	NR	NR	all patients were prescribed oral antibiotics (amoxicillin 1.5 g for 5 days) or clindamycin (1.2 mg for 4 days), and dexamethasone (4 mg for 2 days); rinsing with 0.25% chlorohexidine continued twice a day for 10 days after extraction; naproxen was provided as a nonsteroidal anti-inflammatory drug twice a day
Vettori et al., 2019 [31]	3.2	5.5	51% maxillary, 49% mandibular	15.7% traumatic extractions, 84.3% simple extractions	NR	almost all surgical sites had been sutured, in 10.47% of cases the patient had started an antibiotic therapy before the intervention; after the intervention, the surgeon prescribed antibiotic therapy to 9.23% of patients, steroids to 0.24% of patients, NSAIDs to 3% of patients

Legend: NR, not reported; TID, three times a day; NSAIDs, non-steroidal anti-inflammatory drugs.

## Data Availability

Data are available on request from the corresponding author.
